# Evaluation of C-Reactive Protein and Computer-Aided Analysis of Chest X-rays as Tuberculosis Triage Tests at Health Facilities in Lesotho and South Africa

**DOI:** 10.1093/cid/ciae378

**Published:** 2024-08-27

**Authors:** Shannon Bosman, Irene Ayakaka, Josephine Muhairwe, Mashaete Kamele, Alastair van Heerden, Thandanani Madonsela, Niklaus D Labhardt, Gregor Sommer, Jens Bremerich, Thomas Zoller, Keelin Murphy, Bram van Ginneken, Alfred K Keter, Bart K M Jacobs, Moniek Bresser, Aita Signorell, Tracy R Glass, Lutgarde Lynen, Klaus Reither

**Affiliations:** Centre for Community Based Research, Human Sciences Research Council, Sweetwaters, South Africa; SolidarMed, Partnerships for Health, Maseru, Lesotho; US Agency for International Development (USAID), Washington, DC, USA; SolidarMed, Partnerships for Health, Maseru, Lesotho; Centre for Community Based Research, Human Sciences Research Council, Sweetwaters, South Africa; SAMRC/WITS Developmental Pathways for Health Research Unit, University of the Witwatersrand, Johannesburg, South Africa; Centre for Community Based Research, Human Sciences Research Council, Sweetwaters, South Africa; Division of Clinical Epidemiology, Department of Clinical Research, University Hospital Basel, Basel, Switzerland; University of Basel, Basel, Switzerland; University of Basel, Basel, Switzerland; Department of Radiology, Clinic of Radiology and Nuclear Medicine, University Hospital Basel, Basel, Switzerland; Institute of Radiology and Nuclear Medicine, Hirslanden Klinik St. Anna, Lucerne, Switzerland; University of Basel, Basel, Switzerland; Department of Radiology, Clinic of Radiology and Nuclear Medicine, University Hospital Basel, Basel, Switzerland; Charité—Universitätsmedizin Berlin, corporate member of Freie Universität Berlin and Humboldt–Universität zu Berlin, Department of Infectious Diseases, Respiratory and Critical Care Medicine, Berlin, Germany; Department of Medical Imaging, Radboud University Medical Center, Nijmegen, The Netherlands; Department of Medical Imaging, Radboud University Medical Center, Nijmegen, The Netherlands; Department of Clinical Sciences, Institute of Tropical Medicine, Antwerp, Belgium; Department of Clinical Sciences, Institute of Tropical Medicine, Antwerp, Belgium; University of Basel, Basel, Switzerland; Department of Medicine, Swiss Tropical and Public Health Institute, Allschwil, Switzerland; University of Basel, Basel, Switzerland; Department of Medicine, Swiss Tropical and Public Health Institute, Allschwil, Switzerland; University of Basel, Basel, Switzerland; Department of Medicine, Swiss Tropical and Public Health Institute, Allschwil, Switzerland; Department of Clinical Sciences, Institute of Tropical Medicine, Antwerp, Belgium; University of Basel, Basel, Switzerland; Department of Medicine, Swiss Tropical and Public Health Institute, Allschwil, Switzerland

**Keywords:** tuberculosis, triage test, computer-aided detection, chest X-ray, C-reactive protein

## Abstract

**Background:**

To improve tuberculosis case-finding, rapid, non-sputum triage tests need to be developed according to the World Health Organization target product profile (TPP) (>90% sensitivity, >70% specificity). We prospectively evaluated and compared artificial intelligence–based, computer-aided detection software, CAD4TBv7, and C-reactive protein assay (CRP) as triage tests at health facilities in Lesotho and South Africa.

**Methods:**

Adults (≥18 years) presenting with ≥1 of the 4 cardinal tuberculosis symptoms were consecutively recruited between February 2021 and April 2022. After informed consent, each participant underwent a digital chest X-ray for CAD4TBv7 and a CRP test. Participants provided 1 sputum sample for Xpert MTB/RIF Ultra and Xpert MTB/RIF and 1 for liquid culture. Additionally, an expert radiologist read the chest X-rays via teleradiology. For primary analysis, a composite microbiological reference standard (ie, positive culture or Xpert Ultra) was used.

**Results:**

We enrolled 1392 participants, 48% were people with HIV and 24% had previously tuberculosis. The receiver operating characteristic curve for CAD4TBv7 and CRP showed an area under the curve of .87 (95% CI: .84–.91) and .80 (95% CI: .76–.84), respectively. At thresholds corresponding to 90% sensitivity, specificity was 68.2% (95% CI: 65.4–71.0%) and 38.2% (95% CI: 35.3–41.1%) for CAD4TBv7 and CRP, respectively. CAD4TBv7 detected tuberculosis as well as an expert radiologist. CAD4TBv7 almost met the TPP criteria for tuberculosis triage.

**Conclusions:**

CAD4TBv7 is accurate as a triage test for patients with tuberculosis symptoms from areas with a high tuberculosis and HIV burden. The role of CRP in tuberculosis triage requires further research.

**Clinical Trials Registration:**

Clinicaltrials.gov identifier: NCT04666311.

## Teaser

The evaluation of tuberculosis triage tests in symptomatic patients at healthcare facilities in Lesotho and South Africa showed that CAD4TBv7 was accurate but, while promising, did not fulfill WHO target product profile criteria. The accuracy of C-reactive protein remained unsatisfactory.

## Graphical Abstract

**Graphical Abstract:**
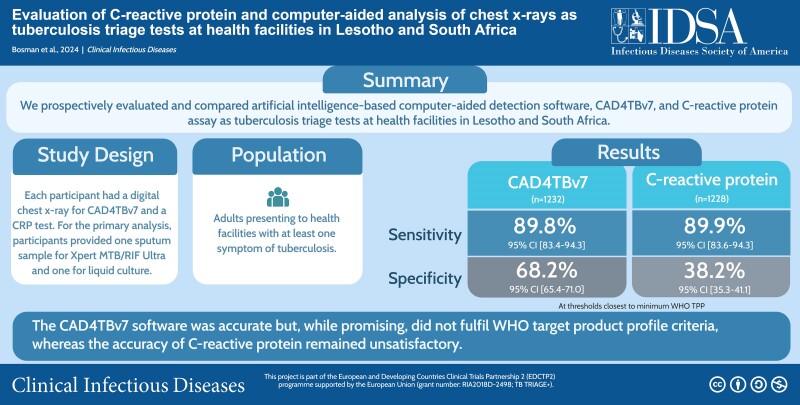
https://tidbitapp.io/tidbits/evaluation-of-c-reactive-protein-and-computer-aided-analysis-of-chest-x-rays-as-tuberculosis-triage-tests-at-health-facilities-in-lesotho-and-south-africa-cd20af86-fe13-42f5-9a51-4733a4788d7b?utm_campaign=tidbitlinkshare&utm_source=ITP

Tuberculosis is a serious threat to global health. There is an urgent need for better detection of this devastating disease [1]. Systematic screening, a provider-initiated search strategy in high-risk or high-prevalence groups, and triage testing, a selection of individuals presenting to healthcare facilities with symptoms or test results, are critical elements in the fight against tuberculosis. According to World Health Organization (WHO) target product profiles (TPPs), a tuberculosis triage tests should be non-sputum, rapid, accurate, inexpensive, and easy to use, with a required minimum sensitivity and specificity of more than 90% and more than 70%, and optimally more than 95% and more than 80%, respectively [2]. Screening and triage strategies should reduce diagnostic costs, offer early access to diagnosis and timely treatment initiation, improve patient outcomes, and ultimately reduce transmission [3, 4].

The WHO conditionally recommends chest X-ray combined with computer-aided detection (CAD) software for tuberculosis screening and triage in individuals aged 15 years and younger [5]. CAD systems show overall good diagnostic accuracy [6, 7] and artificial intelligence (AI) and mobile or portable X-ray systems offer opportunities to use CAD directly in the community [8]. However, it is necessary to establish a context-specific CAD threshold score, based on demographic and epidemiological data, and to consider information on X-ray equipment, programmatic capacity, and available budget when implementing CAD [9–11].

The WHO also conditionally recommends screening for tuberculosis with C-reactive protein (CRP) in people with human immunodeficiency virus (HIV) [5]. CRP is an acute-phase marker for inflammation and infection. Point-of-care test versions enable use at the community level. Most evidence on the diagnostic performance of CRP derives from studies in people with HIV as a tuberculosis screen test [12–17], while data on symptomatic patients who were screened irrespective of HIV status have only recently been collected [18–22].

In this study we investigated the diagnostic performance of 2 potential tuberculosis triage tests, CAD and point-of care CRP testing, in adults presenting with tuberculosis symptoms at health facilities in Southern Africa.

## METHODS

### Study Design

We performed a prospective, multicenter study to evaluate the accuracy of CAD4TBv7 CAD software (Delft Imaging, Netherlands) and the point-of-care CRP test (Afinion AS100 Analyzer, Alere, USA) for triage of tuberculosis at 2 research sites in Lesotho and South Africa as part of the TB TRIAGE+ project. In Lesotho, participants were recruited from the Butha Buthe Government Hospital and in South Africa from 10 primary health care clinics in the Greater Edendale area of Pietermaritzburg, and referred to the research site. This trial was registered at Clinicaltrials.gov (identifier: NCT04666311).

### Participants

Adults (age ≥18 years) with 1 or more cardinal tuberculosis symptoms (cough, fever, night sweats, weight loss) for any duration were eligible to participate. Reasons for exclusion were reported pregnancy, any medical condition that could compromise the participant's well-being by participating in the study, seriously ill participants, and current tuberculosis treatment.

### Procedures

The consecutive enrollment included collection of demographic, anthropometric, medical history data, HIV rapid testing, and physical examination. On the day of enrollment, all participants received a digital posterior-anterior chest X-ray by qualified radiographers for CAD4TBv7 analysis using a FUJIFILM FDR Smart system (FUJIFILM Corporation, Japan) in South Africa and a Delft Light system (Delft Imaging, Netherlands) in Lesotho. CAD4TBv7 software quantifies the probability of pulmonary tuberculosis on a chest X-ray and provides a tuberculosis probability score (0–100). X-rays were securely sent to the University Hospital Basel, Switzerland, where an expert radiologist, blinded to study results, read them within 24–48 hours. Furthermore, a CRP test was performed from capillary blood samples providing continuous values in milligrams per liter.

At enrollment, or within 1 working day, 2 spot sputum samples were collected, 1 was split for onsite Xpert MTB/RIF (Xpert; Cepheid, USA) and onsite Xpert MTB/RIF Ultra (Xpert Ultra) and 1 for liquid culture (BACTEC MGIT 960; Becton Dickinson, USA). Culture was performed at certified, quality-assured, external laboratories (ie, Caprisa [Durban, South Africa] and National Reference Laboratory [Maseru, Lesotho]). Study personnel carrying out reference tests were blinded to the index test results. Determine TB LAM Ag (TB LAM; Abbott, USA) urine testing was performed for people with HIV as part of the advanced HIV disease care package [23]. Participants were asked about vital status and tuberculosis treatment 12 weeks post-enrollment. The study underwent independent remote and onsite monitoring. Data were captured directly on MACRO software (Ennov, France).

### Definition of Reference Standards

The composite microbiological reference standard (CMRS) for the primary analysis was defined as a positive result for either culture or Xpert Ultra. In participants without a history of tuberculosis, trace Xpert Ultra results were considered positive for tuberculosis. Both culture and Xpert Ultra being negative was considered negative for tuberculosis disease. All other cases were considered indeterminate and excluded from the analyses.

The extended composite reference standard (eCRS) for the secondary and exploratory analyses was defined as a positive result on any of the following tests: culture, Xpert Ultra (with trace definition as above), Xpert, TB LAM (for people with HIV), tuberculosis treatment decision based on clinical grounds (signs or symptoms), and reading of the X-ray. We aimed for the highest possible sensitivity for this reference standard by including clinically relevant criteria that reflect clinical practice, although this may overestimate the specificity and underestimate the sensitivity of the index test as not all clinical tuberculosis cases have actually tuberculosis disease.

The reading of X-rays was classified by the expert into 4 categories: highly probable tuberculosis, possible tuberculosis, abnormal but not tuberculosis, and normal. A positive radiological reference standard was defined as highly probable or possible tuberculosis.

### Statistical Analysis

We applied Standards for Reporting Diagnostic accuracy studies (Supplementary Table 1) [24]. Descriptive statistics were used to summarize participant demographic and clinical data. A sample size of 1383 was a priori calculated to reach an acceptable level of precision for sensitivity and specificity estimates of the index cases (assumptions: 10% tuberculosis prevalence, 90% sensitivity, 70% specificity; maximum marginal error of estimate: 0.05; type 1 error: 0.05).

The primary objective was to measure the diagnostic accuracy of CAD4TBv7 and CRP against the CMRS. The receiver operating characteristic (ROC) graph was constructed for each objective. The area under the ROC curve (AUROC), sensitivity, specificity, and positive and negative predictive values for the threshold were reported with their respective 95% confidence intervals (CIs). For both CAD4TBv7 and CRP, we selected the threshold closest to 90% sensitivity.

The expert radiologist's performance was assessed by plotting 3 reader categories as positive results (1: highly probable tuberculosis; 2: highly probable tuberculosis, possible tuberculosis; 3: highly probable tuberculosis, possible tuberculosis, abnormal but not tuberculosis) into the ROC graph.

Prespecified subgroup analyses were performed for gender, age groups, HIV status (positive with CD4 <200 cells/µL, positive with CD4 ≥200 cells/µL, negative), history of tuberculosis, smoking, Alcohol Use Disorders Identification Test (AUDIT-C), body mass index (BMI), and country. Unplanned subgroup analysis compared HIV status according to antiretroviral therapy (ART) (positive on ART, positive not on ART, negative).

The secondary objectives assessed the diagnostic accuracy of CAD4TBv7 and CRP against an eCRS and the single microbiological reference standards (culture, Xpert, and Xpert Ultra). Additionally, CAD4TBv7 was compared with a radiological reference standard.

As an exploratory analysis, we assessed the diagnostic accuracy of the combined triage test of CAD4TBv7 and CRP against the single microbiological reference tests, the CMRS and the eCRS. The combined index test was considered positive if either CAD4TBv7 or CRP was above the study-derived threshold, negative if both tests were below the threshold, or missing and excluded from this analysis.

As an additional exploratory analysis, we calculated, at the thresholds for CAD4TBv7 and CRP, the estimated number of individuals who would need to be tested with a confirmatory test after triaging to detect 1 bacteriologically confirmed case as a proxy for triage capability, as well as the proportion of subsequent confirmatory assays saved as a proxy for cost-effectiveness (a confirmation test for all would be 0%).

A post hoc sensitivity analysis was performed due to considerable variability in time to inoculation of culture. We investigated the effect of this timing (≤3 days vs >3 days) on the diagnostic performance of CAD4TBv7 and CRP. All analyses were performed using Stata (StataCorp, College Station, TX, USA), version 16.1.

### Ethical Considerations

Written informed consent was obtained from study participants before data collection. The Northwest and Central Switzerland Ethics Committee (AO_2022-00014), the National Health Research and Ethics Committee of Lesotho (ID 100-2020), the Human Sciences Research Council Research Ethics Committee (REC 2/23/09/20), and the KwaZulu Natal Provincial Department of Health Research Ethics Committee (KZ_202102_030) in South Africa approved this study.

## RESULTS

Between 15 February 2021 and 5 April 2022, we enrolled 1392 participants: 700 in Lesotho and 692 in South Africa. The median age was 45 years and 54.0% were female. Reported symptoms were cough (91.5%), weight loss (47.6%), night sweats (45.1%), and fever (22.2%). A total of 672 (48.3%) were people with HIV with 84.4% were currently taking ART. Prior history of tuberculosis was reported by 23.8%. In Lesotho, 23.0% of the participants were miners, while in South Africa this was 1.3%. Of participants, 36.4% had a history of tobacco use and 23.9% were at risk of unhealthy alcohol consumption (AUDIT-C score) [25] (Table 1).

**Table 1.** Participant Demographic and Risk Factor Characteristics

	Lesotho (n = 700)	South Africa (n = 692)	Total (n = 1392)
Age			
Age, median (IQR), y	44 (32–60)	46 (33–55)	45 (33–57)
Gender (self-reported)			
Female	377 (53.9%)	375 (54.2%)	752 (54.0%)
Male	323 (46.1%)	317 (45.8%)	640 (46.0%)
Tuberculosis symptoms			
Cough	670 (95.7%)	604 (87.3%)	1274 (91.5%)
Weight loss	359 (51.3%)	304 (43.9%)	663 (47.6%)
Night sweats	301 (43.0%)	327 (47.3%)	628 (45.1%)
Fever	202 (28.9%)	107 (15.5%)	309 (22.2%)
BCG vaccination			
Yes	599 (85.6%)	530 (76.8%)	1129 (81.2%)
No	101 (14.4%)	110 (15.9%)	211 (15.2%)
Unknown/missing	0	52 (7.2%)	52 (3.6%)
Previous tuberculosis			
Yes	122 (17.4%)	209 (30.3%)	331 (23.8%)
No	576 (82.3%)	473 (68.6%)	1049 (75.5%)
Refused/unknown/missing	2 (0.3%)	10 (1.1%)	12 (0.8%)
Risk factors^a^			
Miner	161 (23.0%)	9 (1.3%)	170 (12.2%)
Healthcare worker	13 (1.9%)	16 (2.3%)	29 (2.1%)
History of tobacco use	254 (36.3%)	252 (36.5%)	506 (36.4%)
Risky drinking behavior^b^	205 (29.3%)	127 (18.4%)	332 (23.9%)
HIV status			
People with HIV	335 (47.9%)	337 (48.8%)	672 (48.3%)
HIV negative	357 (51.0%)	336 (48.7%)	693 (49.9%)
Refused testing	8 (1.1%)	17 (2.5%)	25 (1.8%)
CD4 cell count (cells/µL) in people with HIV^c^			
>200	180 (54.2%)	286 (85.1%)	466 (69.8%)
≤200	152 (45.8%)	50 (14.9%)	202 (30.2%)
ART among people with HIV			
Not receiving ART	70 (20.9%)	19 (5.6%)	89 (13.2%)
Receiving ART	265 (79.1%)	302 (89.6%)	567 (84.4%)
Concomitant diseases (reported)			
Diabetes	29 (4.1%)	29 (4.2%)	58 (4.2%)
Hypertension	101 (14.4%)	134 (19.4%)	235 (16.9%)
Heart disease	3 (0.4%)	3 (0.4%)	6 (0.4%)
Kidney disease	1 (0.1%)	9 (1.3%)	10 (0.7%)
Malnutrition	4 (0.6%)	24 (3.5%)	28 (2.0%)
Cancer	0 (0.0%)	4 (0.6%)	4 (0.3%)
Other	0 (0.0%)	18 (2.6%)	18 (1.3%)
Bronchitis	2 (0.3%)	2 (0.3%)	4 (0.3%)
Pneumonia	0 (0.0%)	10 (1.4%)	10 (0.7%)
Bronchial asthma	8 (1.1%)	30 (4.3%)	38 (2.7%)
Silicosis	2 (0.3%)	1 (0.1%)	3 (0.2%)
Other	2 (0.3%)	5 (0.7%)	7 (0.5%)
Clinical examination^d^			
Weight, median (IQR), kg	59 (50–70)	62 (53–74)	60 (52–72)
Body mass index, median (IQR), kg/m^2^	22 (19–27)	22 (19–28)	22 (19–27)
Respiratory rate, median (IQR), breaths/min	18 (16–18)	20 (18–20)	18 (18–20)
Temperature, median (IQR), °C	37 (37–37)	37 (36–37)	37 (37–37)
Edema	13 (1.9%)	8 (1.2%)	21 (1.5%)
Dehydration	2 (0.3%)	48 (7.0%)	50 (3.6%)
Pain/discomfort	5 (0.7%)	135 (19.6%)	140 (10.1%)
Lymph nodes (>1 cm)	2 (0.3%)	2 (0.3%)	4 (0.3%)

Data are presented as n (%) unless otherwise indicated. Missing data are displayed or indicated in the footnotes.

Abbreviations: ART, antiretroviral therapy; AUDIT-C, Alcohol Use Disorders Identification Test; BCG, bacille Calmette Guérin; HIV, human immunodeficiency virus; IQR, interquartile range.

^a^Missing data: miner (n = 2), healthcare worker (n = 2), tobacco use (n = 11), and risky drinking (n = 10).

^b^AUDIT-C score—at risk: men ≥4, women ≥3.

^c^VISITECT CD4 Advanced Disease (AccuBio Limited, Alva, UK).

^d^Missing data: weight and body mass index (n = 3), respiratory rate (n = 8), temperature (n = 6), edema (n = 3), dehydration (n = 3), pain/discomfort (n = 3), and lymph nodes (n = 3).

**Figure 1.**
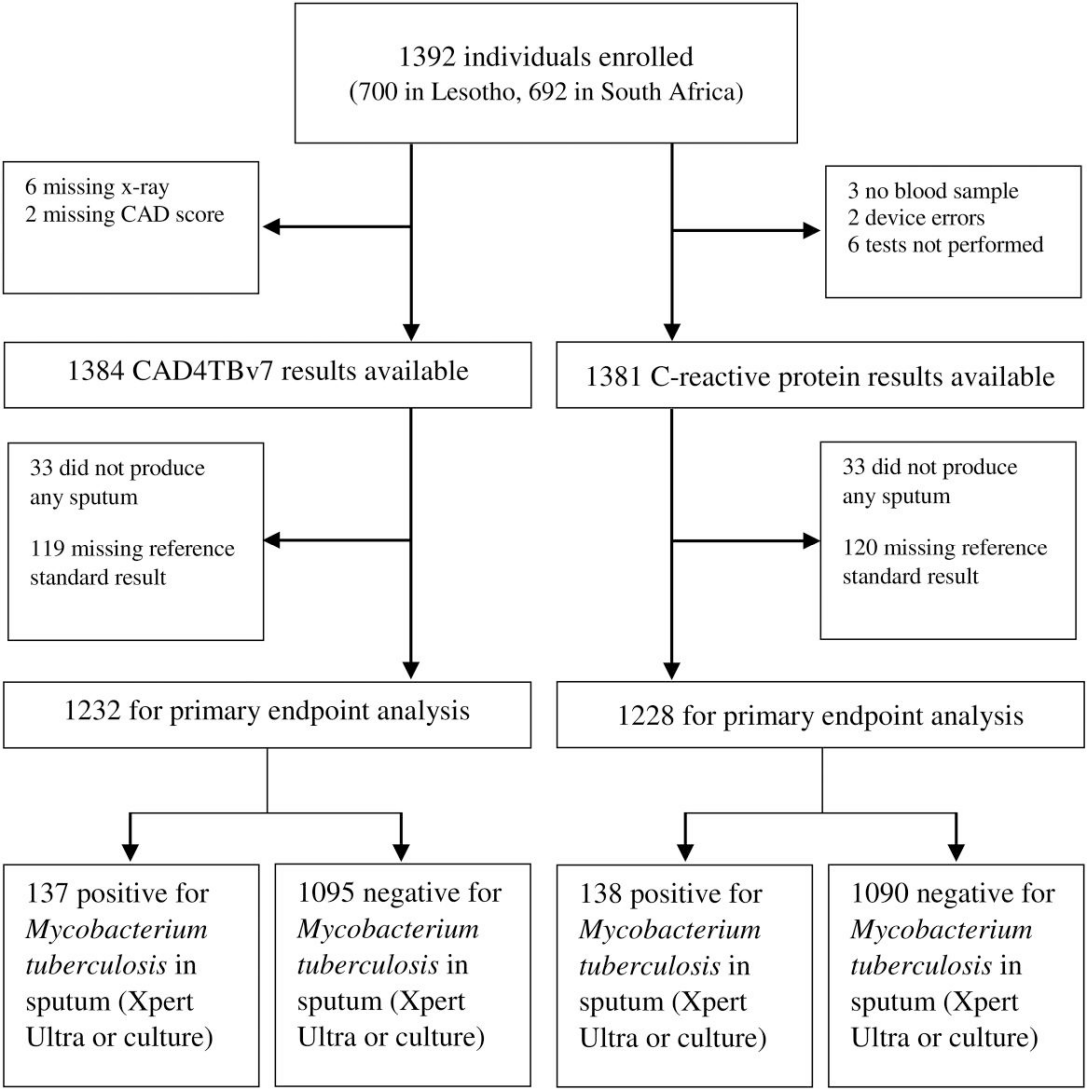
Study flow diagram. Abbreviation: CAD, computer-aided detection.

X-rays with CAD4TBv7 were available in 1384 (99%) and CRP results in 1381 (99%) of the 1392 enrolled participants. Chest X-ray was not performed in 6 participants and CAD4TBv7 scores were not available for 2 participants with an X-ray image. There were 11 CRP results missing, with 3 participants missing a blood sample, an error with the CRP analyzer for 2 participants, and CRP test not being conducted in 6 participants. In total, 33 participants (2.4%) were unable to produce any sputum samples. Missing CMRS results led to 119 and 120 exclusions from the analysis of CAD4TBv7 and CRP, respectively (Figure 1).

Xpert Ultra and culture were available for 1236 (96%) participants. In total, 139 (11%) were tuberculosis positive, 79 (6%) were positive on both Xpert Ultra and culture, 40 (3%) were positive on Xpert Ultra only, and 20 (2%) were positive on culture only. Of the 40 with positive Xpert Ultra results only, 11 (28%) had a history of previous tuberculosis. In 2 patients from Lesotho and 6 patients from South Africa, rifampicin-resistant tuberculosis was detected with Xpert or Xpert Ultra.

**Figure 2.**
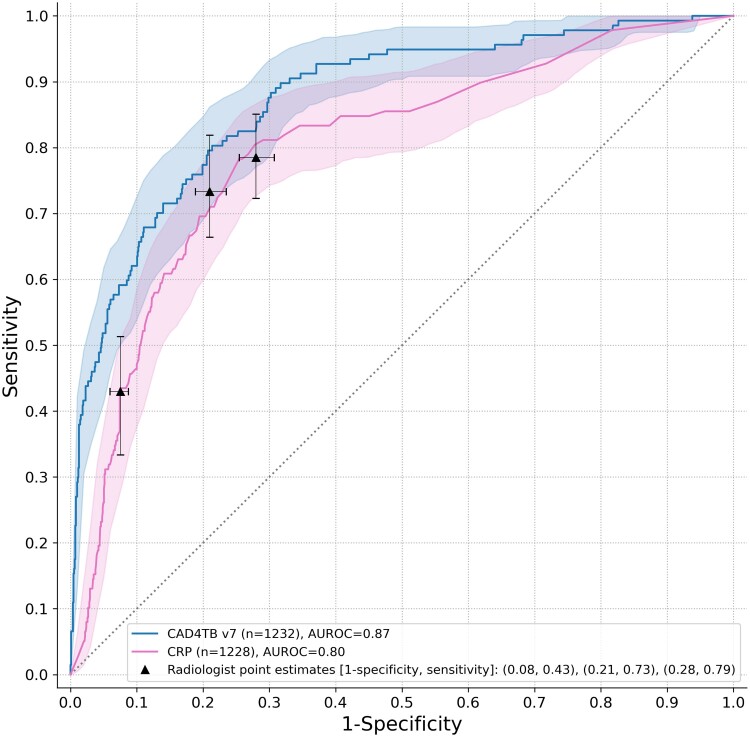
CAD4TBv7, CRP and expert radiologist performance. Composite microbiological reference standard (culture or Xpert Ultra). 95% CIs calculated by bootstrapping. Expert radiologist point estimates are generated by choosing different reading thresholds—that is, cases classified as highly probable tuberculosis (left point estimate), highly probable or possible tuberculosis (middle point estimate), or highly probable or possible tuberculosis or abnormal but not tuberculosis (right point estimate). Abbreviations: AUROC, area under the receiver operating characteristic curve; CI, confidence interval; CRP, C-reactive protein.

The ROC curve for CAD4TBv7 against the CMRS showed an AUROC of .87 (95% CI: .84–.91). The ROC curve for the CRP test against the CMRS showed an AUROC of .80 (95% CI: .76–.84) (Figure 2).

The threshold closest to the minimum WHO TPP was set for CAD4TBv7 at 27, with 89.8% sensitivity (95% CI: 83.4–94.3%), 68.2% specificity (95% CI: 65.4–71.0%), 26.1% positive predictive value (95% CI: 22.2–30.3%), and 98.2% negative predictive value (95% CI: 96.9–99.0%). The CRP threshold was determined at 7 mg/L with 89.9% sensitivity (95% CI: 83.6–94.3%), 38.2% specificity (95% CI: 35.3–41.1%), 15.5% positive predictive value (95% CI: 13.1–18.2%), and 96.7% negative predictive value (95% CI: 94.6–98.2%).

Sensitivity and specificity for human radiological assessment according to the 3 reader categories (highly probable tuberculosis, highly probable or possible tuberculosis, highly probable or possible tuberculosis or abnormal but not tuberculosis) was 43% and 92%, 73% and 79%, and 79% and 72%, respectively (Figure 2).

Within subgroups, CAD4TBv7 and CRP showed a variable performance at the study-derived threshold when compared against CMRS (eg, both tests performed poorly in participants with high BMI) (Table 2). The AUROC for CAD4TBv7 differed significantly in participants with and without history of tuberculosis (*P* = .02) and if they were recruited in Lesotho or South Africa (*P* = .04) (Supplementary Table 2).

**Table 2.** CAD4TBv7 and CRP Performance at the Study-Derived Threshold in All Participants and by Subgroups Against a Composite Microbiological Reference Standard

	n	Sensitivity, %	95% CI	Specificity, %	95% CI
CAD4TBv7					
All	1232	89.8	83.4–94.3	68.2	65.4–71.0
Female	679	94.6	87.9–98.2	57.7	53.6–61.7
Male	553	79.5	64.7–90.2	80.4	76.6–83.7
18–34 y	381	89.5	78.5–96.0	82.7	78.2–86.7
35–64 y	668	89.4	79.4–95.6	64.8	60.8–68.6
≥65 y	161	90.9	58.7–99.8	52.7	44.4–60.9
HIV negative	610	89.3	78.1–96.0	70.6	66.6–74.3
PWH, CD4 >200 cells/µL	410	86.1	70.5–95.3	71.4	66.5–75.9
PWH, CD4 ≤200 cells/µL	188	95.1	83.5–99.4	49.0	40.7–57.3
HIV negative^a^	610	89.3	78.1–96.0	70.6	66.6–74.3
PWH, not on ART^a^	104	87.5	71.0–96.5	61.1	48.9–72.4
PWH, on ART^a^	518	91.8	80.4–97.7	66.5	62.1–70.8
History of tuberculosis	290	92.6	75.7–99.1	46.8	40.6–53.0
No history of tuberculosis	933	89.0	81.6–94.2	75.1	72.0–78.0
History of smoking	463	90.9	81.3–96.6	59.2	54.2–64.1
Never smoked	763	88.4	78.4–94.9	73.2	69.7–76.5
Risky alcohol drinking (AUDIT-C)^b^	304	89.3	71.8–97.7	67.0	61.1–72.5
No history of alcohol use (AUDIT-C)^b^	928	89.9	82.7–94.9	68.6	65.3–71.8
BMI^c^ <18.5 kg/m^2^	262	95.2	86.7–99.0	49.7	42.6–56.9
BMI^c^ 18.5–24.9 kg/m^2^	566	91.1	80.4–97.0	66.5	62.2–70.6
BMI^c^ ≥25.0 kg/m^2^	404	66.7	41.0–86.7	80.1	75.7–83.9
Lesotho	683	90.1	80.7–95.9	61.4	57.5–65.3
South Africa	549	89.4	79.4–95.6	76.8	72.8–80.5
CRP					
All	1228	89.9	83.6–94.3	38.2	35.3–41.1
Female	678	92.5	85.1–96.9	37.8	33.8–41.8
Male	550	84.4	70.5–93.5	38.6	34.3–43.0
18–34 y	381	89.7	78.8–96.1	44.6	39.1–50.2
35–64 y	665	87.9	77.5–94.6	34.6	30.8–38.5
≥65 y	160	100	71.5–100	38.3	30.4–46.6
HIV negative	611	84.5	72.6–92.7	38.7	34.6–42.9
PWH, CD4 >200 cells/µL	406	88.6	73.3–96.8	42.0	37.0–47.3
PWH, CD4 ≤200 cells/µL	187	100	91.4–100	26.7	19.7–34.7
HIV negative^a^	611	84.5	72.6–92.7	38.7	34.6–42.9
PWH, not on ART^a^	103	96.8	83.3–99.9	26.4	16.7–38.1
PWH, on ART^a^	514	91.8	80.4–97.7	39.4	34.9–44.0
History of tuberculosis	290	78.6	59.0–91.7	37.8	31.9–44.0
No history of tuberculosis	929	92.7	86.0–96.8	38.4	35.1–41.8
History of smoking	463	92.4	83.2–97.5	40.8	35.9–45.8
Never smoked	759	87.1	77.0–93.9	36.9	33.3–40.6
Risky alcohol drinking (AUDIT-C)^b^	305	92.9	76.5–99.1	43.3	37.4–49.4
No history of alcohol use (AUDIT-C)^b^	923	89.1	81.7–94.2	36.4	33.1–39.8
BMI^c^ <18.5 kg/m^2^	262	95.2	86.7–99.0	36.7	30.0–43.8
BMI^c^ 18.5–24.9 kg/m^2^	562	90.9	80.0–97.0	43.0	38.6–47.4
BMI^c^ ≥25.0 kg/m^2^	404	70.0	45.7–88.1	32.6	27.9–37.5
Lesotho	676	94.4	86.4–98.5	36.6	32.7–40.5
South Africa	549	84.8	73.9–92.5	40.2	35.8–44.7

Abbreviations: AUDIT-C, Alcohol Use Disorders Identification Test; BCG, bacille Calmette Guérin; BMI, body mass index; CI, confidence interval; CRP, C-reactive protein; HIV, human immunodeficiency virus; PWH, persons with HIV.

^a^This subgroup analysis was not planned.

^b^AUDIT-C score—at risk: men ≥4, women ≥3.

^c^BMI (underweight [<18.5 kg/m^2^], normal weight [18.5–24.9 kg/m^2^], overweight or obese [≥25.0 kg/m^2^]).

The AUROC values analyzed against the single reference standards (culture, Xpert, Xpert Ultra) and the eCRS for CAD4TBv7 ranged from .86 to .91. The AUROC for CAD4TBv7 was .90 (95% CI: .88–.92) against the radiological reference standard. For CRP, the AUROC against the single reference standards were in a similar range (.82–.83), while the AUROC was lower when compared with the eCRS (.70) (Table 3).

**Table 3.** CAD4TBv7 and CRP Performance in all Participants Against Single and Against Extended Composite Reference Standards

Index Tests	Reference Standards	AUROC	95% CI
CAD4TBv7 (n = 1232)	Culture (n = 1236)	.87	.83–.91
	Xpert (n = 1331)	.91	.88–.94
	Xpert Ultra (n = 1334)	.90	.88–.93
	Extended composite reference standard (n = 1266)	.86	.83–.88
	Expert radiologist (n = 1362)	.90	.88–.92
CRP (n = 1228)	Culture (n = 1327)	.82	.78–.87
	Xpert (n = 1327)	.83	.78–.87
	Xpert Ultra (n = 1331)	.82	.78–.86
	Extended composite reference standard (n = 1262)	.70	.67–.73

Abbreviations: AUROC, area under the receiver operating characteristic curve; CI, confidence interval; CRP, C-reactive protein.

A post hoc, head-to-head comparison of CAD4TBv7 and CRP showed that the difference between the 2 modalities is statistically significant (McNemar's test for paired samples, *P* < .001).

The evaluation of the combination of CAD4TBv7 and CRP against single reference standards, CMRS, or eCRS showed a high sensitivity (sensitivity: between 90% and 98%) at the expense of a lower specificity across all analyses (specificity: between 30% and 38%) (Table 4).

**Table 4.** Diagnostic Accuracy of the Combination of CAD4TBv7 and CRP at Their Study-Derived Thresholds Compared With Single Reference Standards, Composite Microbiological Reference Standard, and the Extended Composite Reference Standard

Reference Standards	Combination of CAD4TB and CRP			
	Sensitivity, %	95% CI	Specificity, %	95% CI
Culture alone (n = 1240)	94.8	88.4–98.3	30.1	27.4–32.9
Xpert alone (n = 1335)	98.1	93.4–99.8	31.3	28.7–33.9
Xpert Ultra alone (n = 1339)	96.6	91.6–99.1	31.2	28.6–33.9
Composite microbiological reference standard (n = 1236)	95.6	90.7–98.4	30.8	28.0–33.6
Extended composite reference standard (n = 1263)	89.7	86.4–92.4	36.5	33.2–39.9

Abbreviations: CI, confidence interval; CRP, C-reactive protein.

**Figure 3.**
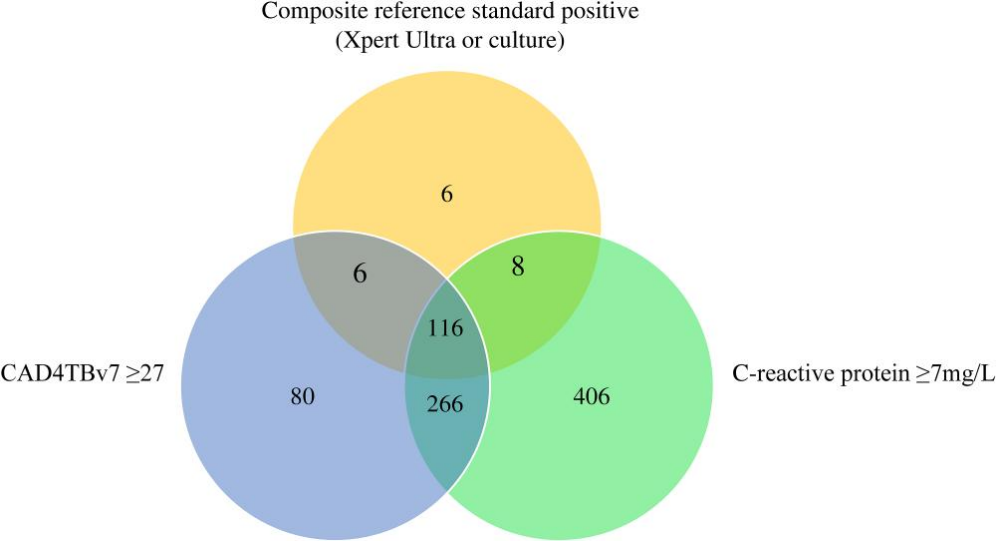
Venn diagram of participants with positive composite reference standard and CAD4TBv7 and CRP above the threshold. Only participants with results from all 3 tests are shown. Abbreviation: CRP, C-reactive protein.

In the Venn diagram, CAD4TBv7 and CRP overlap in 116 of 136 true-positive cases, while CAD4TBv7 alone identified 6 true-positive cases not detected by CRP, and CRP identified 8 cases not detected by CAD4TBv7 (Figure 3).

The number needed to test after triaging to identify 1 microbiologically confirmed case was 4 for CAD4TBv7 (95% CI: 3–5) and 6 for CRP (95% CI: 5–8). In a triage scenario, CAD4TBv7 would have avoided 62% (95% CI: 59–64%) and CRP 35% (95% CI: 33–38%) of confirmatory tests compared with an Xpert Ultra-for-all scenario.

For the post hoc sensitivity analysis, we conducted an analysis against the CMRS standard including only those culture samples processed in 3 days or fewer. The AUROC for this subgroup was .84 (95% CI: .78–.90) and .80 (95% CI: .74–.86) for CAD4TBv7 and CRP, respectively.

In total, 37 serious adverse events were reported, 23 in Lesotho and 14 in South Africa, none of which was considered related to the investigational devices or study procedures.

## DISCUSSION

This is the first prospective, multicenter simultaneous evaluation and comparison of CAD for digital chest radiography and CRP in patients with presumptive tuberculosis at health facilities in Southern Africa.

The key study finding is that CAD4TBv7, evaluated against a CMRS, is close to the minimum sensitivity and specificity as defined by WHO's TPP for a community-based triage or referral tests with a high negative predictive value. On the other hand, CRP does not meet TPP criteria, showing a sensitivity of almost 90% and a specificity of only 38%.

The AUROC for CAD4TBv7 in our study was almost identical to the one in a facility-based screening study from Bangladesh with Xpert as the only reference standard [6]. In a community-based, multimorbidity study in KwaZulu-Natal, CAD4TBv7 showed a lower diagnostic performance, with an AUROC of .80, against the CMRS (culture and Xpert Ultra) [26]. However, the study design could be prone to bias as CAD4TBv7 was re-analyzed using sputum results collected only if an older, less accurate CAD4TB version was above a certain threshold. Consistent with previous CAD studies [11, 27, 28], the AUROC of CAD4TBv7 was significantly lower in individuals with previous tuberculosis, most likely due to post-tuberculous residual lung abnormalities resulting in lower specificity.

Using a radiological reference standard, the AUROC for CAD4TBv7 remained high at .90, although the performance compared with the radiologist reference standard was not as good as that previously reported for CAD4TBv6 in a study with a different expert panel [29]. CAD4TBv7 performed as well as an expert radiologist, comparable to results of a study on CAD4TBv6 [29], but not to 1 study on CAD4TBv7 [6], which reported a superiority of the software; this may be explained by differences in radiological expertise.

Recently published facility-based studies assessing the diagnostic accuracy of CRP, which included, as in our study, people with and without HIV, had various combinations of reference standards and reported at a threshold of 10 mg/L similar sensitivities and specificities (ie, 77.7% and 66.6%, 93% and 54%, 86.6% and 34.8%, respectively) [18–20]. These findings are in line with results from a meta-analysis of the accuracy of CRP for active pulmonary tuberculosis in outpatients [13] and similar to community studies with a threshold of 5 mg/L [21, 30]. We did not find significant differences in AUROC for CRP by HIV status or CD4 count strata, in contrast to previously reported results from people with HIV initiating ART, showing TPP compliance [17].

Combining CAD4TBv7 and CRP using their study-derived thresholds increased the sensitivity with a substantial sacrifice in specificity, which speaks against a simple combination of the 2 tests. Potentially interesting could be a combination where CRP is used as a second triage test instead of an immediate Xpert Ultra and to test only in a specific range of the CAD4TBv7 score, an approach currently being evaluated in a large-scale community study [31].

In our study CAD4TBv7 performed better than CRP in terms of healthcare-relevant performance indicators—that is, the number of post-triage confirmatory tests required to identify a confirmed tuberculosis case and the number of avoided confirmatory tests—to give an indication of the triage capacity and cost-effectiveness of the 2 triage candidates.

This study has potential limitations. Despite clear instructions, the processing time of sputum for culture varied in both external laboratories, a potential reason for compromised mycobacterial growth. The post hoc sensitivity analysis showed, however, only a small impact on the AUROC value for CAD4TBv7, with slightly lower values for an incubation time of 3 days or less compared with the whole study population. Furthermore, different X-ray equipment in Lesotho and South Africa may have contributed to the site-specific differences in CAD4TBv7 performance. There is also a possible bias due to participants with previous tuberculosis who were Xpert Ultra–positive and culture-negative, as this may have led to overestimating sensitivity and underestimating specificity of CAD4TBv7, but not CRP. Another limitation was that only 1 radiologist analyzed the images, while previous studies have shown some relevant differences between human readers.

In summary, CAD4TBv7 narrowly missed the WHO TPP target, suggesting that CAD4TBv7 might still have potential as a triage approach for people with presumptive tuberculosis at the facility level. In our study, the latest version of this AI-powered software detects tuberculosis as reliably as an expert radiologist, but within seconds, and avoids almost two-thirds of confirmatory sputum tests compared with testing all with Xpert Ultra. However, various barriers might challenge wider adoption of CAD, with developing simplified procedures for local threshold selection and thorough cost-effectiveness analyses being among the most important. The results of our study disqualify CRP as a stand-alone triage for tuberculosis. However, as the test has low costs and performs unaffected by HIV status, CRP might have potential clinical utility in ruling out tuberculosis in settings or facilities where other diagnostic tests are not available or affordable [19, 32]. Future research is needed to evaluate the diagnostic value of CRP for tuberculosis triaging, specifically combining it with CAD for chest X-ray [31], symptom screening [15, 21, 30], or promising biomarkers [33].

## Supplementary Data

Supplementary materials are available at *Clinical Infectious Diseases* online. Consisting of data provided by the authors to benefit the reader, the posted materials are not copyedited and are the sole responsibility of the authors, so questions or comments should be addressed to the corresponding author.

## Supplementary Material

ciae378_Supplementary_Data

## Notes

***Author contributions*.** K. R., L. L., B. K. M. J., and T. R. G. conceived the study. K. R., J. M., A. v. H., N. D. L., A. S., B. v. G., B. K. M. J., and T. R. G. designed the study. S. B., I. A., J. M., M. K., and T. M. recruited participants and collected data. T. R. G. and A. K. K. analyzed the data. M. B. was responsible for data management, G. S. and J. B. for X-ray reading, T. Z. for medical monitoring, A. S. for project management, and K. M. for CAD analyses. S. B., K. R., and T. R. G. wrote the first draft of the manuscript and interpreted data. All authors reviewed the draft manuscript, and all contributed to and approved the final manuscript.

***Acknowledgments*.** The authors thank the TB TRIAGE+ study teams and all study participants in Lesotho and South Africa. They are grateful to the members of the TB TRIAGE+ Scientific Advisory Board (A. van’t Hoog (in memoriam), G. Meintjes, C. Yoon, S. Floyd) for their guidance and advice.

***Disclaimer.*** The funders of the study had no role in study design, data collection, data analysis, data interpretation, or writing of the report.

***Data sharing*.** Pseudonymized datasets will be made available on a noncommercial data platform upon reasonable request to the corresponding author.

***Financial support*.** This project is part of the European and Developing Countries Clinical Trials Partnership 2 (EDCTP2) program supported by the European Union (grant number: RIA2018D-2498; TB TRIAGE+).
