# Supplementary material for: Evaluation of C-Reactive Protein and Computer-Aided Analysis of Chest X-rays as Tuberculosis Triage Tests at Health Facilities in Lesotho and South Africa
Source: Clin Infect Dis. 2024 Aug 27;79(5):1293–302. doi: 10.1093/cid/ciae378 (PMC11581699; doi:10.1093/cid/ciae378)
Supplement: ciae378_Supplementary_Data [file ciae378_supplementary_data.docx]

**Supplementary material:**

**Table S1: STARD checklist**

|  | **Section & Topic** | **No** | **Item** | **Reported on page #** |
| --- | --- | --- | --- | --- |
|  |  |  |  |  |
|  | **TITLE OR ABSTRACT** |  | **Evaluation of C-reactive protein and computer-aided analysis of chest x-rays as tuberculosis triage tests at health facilities in Lesotho and South Africa** |  |
|  |  | **1** | Identification as a study of diagnostic accuracy using at least one measure of accuracy  (such as sensitivity, specificity, predictive values, or AUC) | 2 |
|  | **ABSTRACT** |  |  |  |
|  |  | **2** | Structured summary of study design, methods, results, and conclusions  (for specific guidance, see STARD for Abstracts) | 2 |
|  | **INTRODUCTION** |  |  |  |
|  |  | **3** | Scientific and clinical background, including the intended use and clinical role of the index test | 3-4 |
|  |  | **4** | Study objectives and hypotheses | 4 |
|  | **METHODS** |  |  |  |
|  | *Study design* | **5** | Whether data collection was planned before the index test and reference standard  were performed (prospective study) or after (retrospective study) | 4,5 |
|  | *Participants* | **6** | Eligibility criteria | 4 |
|  |  | **7** | On what basis potentially eligible participants were identified  (such as symptoms, results from previous tests, inclusion in registry) | 4 |
|  |  | **8** | Where and when potentially eligible participants were identified (setting, location and dates) | 4 |
|  |  | **9** | Whether participants formed a consecutive, random or convenience series | 4 |
|  | *Test methods* | **10a** | Index test, in sufficient detail to allow replication | 4,5 |
|  |  | **10b** | Reference standard, in sufficient detail to allow replication | 5 |
|  |  | **11** | Rationale for choosing the reference standard (if alternatives exist) | 5 |
|  |  | **12a** | Definition of and rationale for test positivity cut-offs or result categories  of the index test, distinguishing pre-specified from exploratory | 6 |
|  |  | **12b** | Definition of and rationale for test positivity cut-offs or result categories  of the reference standard, distinguishing pre-specified from exploratory | 6 |
|  |  | **13a** | Whether clinical information and reference standard results were available  to the performers/readers of the index test | 5 |
|  |  | **13b** | Whether clinical information and index test results were available  to the assessors of the reference standard | 5 |
|  | *Analysis* | **14** | Methods for estimating or comparing measures of diagnostic accuracy | 6 |
|  |  | **15** | How indeterminate index test or reference standard results were handled | 6 |
|  |  | **16** | How missing data on the index test and reference standard were handled | 6 |
|  |  | **17** | Any analyses of variability in diagnostic accuracy, distinguishing pre-specified from exploratory | 6,7 |
|  |  | **18** | Intended sample size and how it was determined | 6 |
|  | **RESULTS** |  |  |  |
|  | *Participants* | **19** | Flow of participants, using a diagram | 17 |
|  |  | **20** | Baseline demographic and clinical characteristics of participants | 14 |
|  |  | **21a** | Distribution of severity of disease in those with the target condition | 7,8,9,15,16 |
|  |  | **21b** | Distribution of alternative diagnoses in those without the target condition | 14 |
|  |  | **22** | Time interval and any clinical interventions between index test and reference standard | 5 |
|  | *Test results* | **23** | Cross tabulation of the index test results (or their distribution)  by the results of the reference standard | 9 |
|  |  | **24** | Estimates of diagnostic accuracy and their precision (such as 95% confidence intervals) | 9 |
|  |  | **25** | Any adverse events from performing the index test or the reference standard | 9 |
|  | **DISCUSSION** |  |  |  |
|  |  | **26** | Study limitations, including sources of potential bias, statistical uncertainty, and generalisability | 11 |
|  |  | **27** | Implications for practice, including the intended use and clinical role of the index test | 11,12 |
|  | **OTHER INFORMATION** |  |  |  |
|  |  | **28** | Registration number and name of registry | 4 |
|  |  | **29** | Where the full study protocol can be accessed | - |
|  |  | **30** | Sources of funding and other support; role of funders | 13 |
|  |  |  |  |  |

**Table S2: CAD4TBv7 and CRP performance (AUROC) in all participants and by subgroups against a composite microbiological reference standard**

|  |  |  | **AUROC** | **95% CI** | **p-value*** |
| --- | --- | --- | --- | --- | --- |
| **CAD4TBv7** | **All** | **n=1232** | **0.87** | **0.84-0.91** | **-** |
|  | Female | n=679 | 0.88 | 0.84-0.92 | 0.67 |
|  | Male | n=553 | 0.86 | 0.79-0.92 |  |
|  | 18-34 years | n=381 | 0.91 | 0.88-0.97 | 0.05 |
|  | 35-64 years | n=668 | 0.85 | 0.79-0.90 |  |
|  | > 65 years | n=161 | 0.83 | 0.71-0.95 |  |
|  | HIV negative | n=610 | 0.89 | 0.83-0.95 | 0.35 |
|  | PWH, CD4>200 | n=410 | 0.85 | 0.79-0.92 |  |
|  | PWH, CD4≤200 | n=188 | 0.83 | 0.76-0.90 |  |
|  | HIV negative^1^ | n=610 | 0.89 | 0.83-0.95 | 0.53 |
|  | PWH, not on ART^1^ | n=104 | 0.85 | 0.77-0.92 |  |
|  | PWH, on ART^1^ | n=518 | 0.85 | 0.80-0.91 |  |
|  | History of tuberculosis | n=290 | 0.79 | 0.70-0.87 | 0.02 |
|  | No history of tuberculosis | n=933 | 0.90 | 0.86-0.93 |  |
|  | History of smoking | n=463 | 0.87 | 0.82-0.91 | 0.95 |
|  | Never smoked | n=763 | 0.87 | 0.82-0.92 |  |
|  | Risky alcohol drinking (Audit-C)^2^ | n=304 | 0.88 | 0.82-0.94 | 0.85 |
|  | No history of alcohol use (Audit-C)^2^ | n=928 | 0.87 | 0.83-0.91 |  |
|  | BMI^3^ <18.5 kg/m² | n=262 | 0.84 | 0.79-0.90 | 0.31 |
|  | BMI^3^ 18.5-24.9 kg/m² | n=566 | 0.88 | 0.83-0.92 |  |
|  | BMI^3^ ≥25.0 kg/m² | n=404 | 0.76 | 0.64-0.91 |  |
|  | Lesotho | n=683 | 0.84 | 0.79-0.89 | 0.04 |
|  | South Africa | n=549 | 0.91 | 0.87-0.95 |  |
| **CRP** | **All** | **n=1228** | **0.80** | **0.76-0.84** | **-** |
|  | Female | n=678 | 0.81 | 0.76-0.86 | 0.27 |
|  | Male | n=550 | 0.76 | 0.68-0.84 |  |
|  | 18-34 years | n=381 | 0.81 | 0.74-0.87 | 0.07 |
|  | 35-64 years | n=665 | 0.78 | 0.72-0.84 |  |
|  | > 65 years | n=160 | 0.88 | 0.82-0.94 |  |
|  | HIV negative | n=611 | 0.76 | 0.69-0.83 | 0.55 |
|  | PWH, CD4>200 | n=406 | 0.81 | 0.73-0.89 |  |
|  | PWH, CD4≤200 | n=187 | 0.80 | 0.74-0.87 |  |
|  | HIV negative^1^ | n=611 | 0.76 | 0.69-0.83 | 0.44° |
|  | PWH, not on ART^1^ | n=103 | 0.79 | 0.70-0.88 |  |
|  | PWH, on ART^1^ | n=514 | 0.82 | 0.76-0.88 |  |
|  | History of tuberculosis | n=290 | 0.71 | 0.60-0.82 | 0.07 |
|  | No history of tuberculosis | n=929 | 0.82 | 0.78-0.87 |  |
|  | History of smoking | n=463 | 0.80 | 0.74-0.86 | 0.67 |
|  | Never smoked | n=759 | 0.78 | 0.72-0.84 |  |
|  | Risky alcohol drinking (Audit-C)^2^ | n=305 | 0.86 | 0.77-0.91 | 0.29 |
|  | No history of alcohol use (Audit-C)^2^ | n=923 | 0.79 | 0.74-0.84 |  |
|  | BMI^3^ <18.5 kg/m² | n=262 | 0.82 | 0.76-0.88 | 0.18 |
|  | BMI^3^ 18.5-24.9 kg/m² | n=562 | 0.78 | 0.72-0.84 |  |
|  | BMI^3^ ≥25.0 kg/m² | n=404 | 0.67 | 0.52-0.82 |  |
|  | Lesotho | n=676 | 0.82 | 0.77-0.87 | 0.28 |
|  | South Africa | n=549 | 0.77 | 0.71-0.84 |  |

* p-value provides the test of the hypothesis that the AUROC for all subgroups are equal. P-values smaller than 0.05 suggest that at least one of the categories has a statistically significantly different AUROC than the other(s).

^1^ This subgroup analysis was not planned.

^2^ Audit-C score - at risk: men ≥4, women ≥3.

^3^ BMI= body mass index (underweight [<18.5 kg/m²], normal weight [18.5-24.9 kg/m²], overweight or obese [≥25.0 kg/m²]).
